# Cooperative Effect of ZIF-67-Derived Hollow NiCo-LDH and MoS_2_ on Enhancing the Flame Retardancy of Thermoplastic Polyurethane

**DOI:** 10.3390/polym14112204

**Published:** 2022-05-29

**Authors:** Yi Qian, Wenyuan Su, Long Li, Rongmin Zhao, Haoyan Fu, Jiayin Li, Peidong Zhang, Qingjie Guo, Jingjing Ma

**Affiliations:** 1 College of Chemical Engineering, Qingdao University of Science and Technology, Qingdao 266042, China; suwenyuan1226@163.com (W.S.); fuhaoyanfhy@163.com (H.F.); qj_guo@yahoo.com (Q.G.); 2 College of Environment and Safety Engineering, Qingdao University of Science and Technology, Qingdao 266042, China; lijiayin085@163.com (J.L.); 02793@qust.edu.cn (P.Z.); 3 Qingdao University of Science and Technology Library, Qingdao University of Science and Technology, Qingdao 266042, China; zhaorongmin@qust.edu.cn; 4 State Key Laboratory of High-Efficiency Utilization of Coal and Green Chemical Engineering, Ningxia University, Yinchuan 750021, China; majingjing@nxu.edu.cn

**Keywords:** layered double hydroxide, molybdenum disulfide, thermoplastic polyurethane, flame retardancy

## Abstract

In this work, a novel three-dimensional (3D) hollow nickel-cobalt layered double hydroxide (NiCo-LDH) was synthesized using zeolitic imidazole framework-67 (ZIF-67) as a template, and then utilized to functionalize molybdenum disulfide (NiCo-LDH/MoS_2_) via electrostatic force. Flame retardant thermoplastic polyurethane (TPU) composites were prepared by the melt blending method. Compared to pure TPU, NiCo-LDH/MoS_2_ filled TPU composite was endowed with a decrease of 30.9% and 55.7% of the peak heat release rate (PHRR) and the peak smoke production rate (PSPR), respectively. Furthermore, the addition of NiCo-LDH/MoS_2_ can significantly improve the thermal stability and char yield of the TPU composite. The catalytic carbonization effect and dilution effect of NiCo-LDH, and the barrier effect of MoS_2_ nanosheets enable TPU composites with excellent flame retardancy and toxic gas suppression ability.

## 1. Introduction

As an important engineering thermoplastic, thermoplastic polyurethane (TPU) has been widely used in the fields of cable, automotive, building and medical devices because of its good tensile strength, superior mechanical properties, excellent chemical stability and adjustable flexibility [1,2,3,4]. Nevertheless, TPU itself is flammable and releases a large amount of black smoke and toxic gases during the combustion process, posing a great threat to human life and property safety [5]. Therefore, it is highly desirable to find efficient and halogen-free flame retardants to improve the flame retardant and smoke suppression properties of TPU. In recent years, nanoscale fillers such as molybdenum disulfide (MoS_2_) [6], layered double hydroxide (LDH) [7] and graphene (GO) [8] have been used as flame retardant additives for TPU and other polymers.

Layered double hydroxide (LDH) is a layered material similar to brucite, also known as hydrotalcite or anionic clay, mainly composed of a positively charged layer and interlayer anion [9]. The two-dimensional (2D) layered structure of LDH can not only hinder the heat and mass transfer between the combustion zone and the polymer matrix, but also dilute the flammable gases by releasing water vapor and carbon dioxide during the polymer decomposition process [10]. Nevertheless, LDH with higher surface energy tends to aggregate together, which is not conducive to improving the flame retardancy of the polymer [11]. Taking advantage of the difference in internal and external stability of metal-organic frameworks (MOFs) as in situ sacrificial templates to prepare three-dimensional (3D) hollow LDH materials is a dramatic research orientation [12,13]. The construction of hierarchical 3D architectures based on 2D LDH can effectively inhibit the aggregation of LDH nanosheets, thereby improving the compatibility of LDH and polymer matrix [14]. Zeolitic imidazole framework-67 (ZIF-67) has been shown to be ideal sacrificial template for the construction of hollow LDH [15]. Zhang [16] et al. prepared 3D HGM@LDH@DOPO hybrid material using ZIF-67 as in situ sacrificial templates, hollow glass microspheres (HGM) and 9, 10-dihydro-9-oxa-10-phosphaphenanthrene 10-oxide (DOPO) as synergistic flame retardants. The obtained HGM@LDH@DOPO was added to the epoxy (EP) to prepare PPcomposites. The cone calorimeter test (CCT) results showed that the peak heat release rate (PHRR) and total heat release (THR) of EP composites were reduced by 56.4% and 14.7% with 5 wt% HGM@LDH@DOPO loadings, respectively. Typically, the loading level of LDH in polymer composites is relatively low (less than 5%). In order to achieve flame retardant requirements, LDH can be combined with other flame retardants to obtain the synergistic flame retardant effect [17].

As a rising two-dimensional material, molybdenum disulfide (MoS_2_) has excellent mechanical properties, low thermal conductivity and non-combustibility, this facilitates its application as a heat stabilizer and flame retardant in polymer composites [18]. However, the inert surface and interlayer van der Waals forces of MoS_2_ nanosheets easily lead to weak interfacial interaction between MoS_2_ and the polymer matrix, resulting in poor mechanical and flame retardant properties of polymer composites [19]. Therefore, it is necessary to exfoliate MoS_2_ into a single layer or few layers for the purpose of uniform dispersion in the polymer. Peng [20] et al. utilized carboxyl-rich poly(ionic liquid)-PCMVIm to exfoliate and non-covalently functionalize MoS_2_, and employed as nanofillers for polyacrylonitrile (PAN) fiber. The results showed that the tensile strength and elongation at break of the PCMVIm-MoS_2_/PAN composite fiber increased by 55% and 70.9%, respectively. In addition, the peak heat release rate, peak smoke release rate and peak CO generation rate of PCMVIm-MoS_2_/PAN composite fiber decreased by 48.7%, 51.4% and 63.5%, respectively. Hence, the combination of 3D hollow LDH and exfoliated MoS_2_ nanosheets is expected to play a great role in improving the flame retardancy and smoke suppression of TPU.

In this work, the novel 3D hollow NiCo-LDH was synthesized by hydrothermal method using ZIF-67 as in situ sacrificial templates, and the 3D hollow NiCo-LDH and MoS_2_ were hybridized to synthesize 3D hollow NiCo-LDH/MoS_2_ hybrid material. Simultaneously, the structure and morphology of the NiCo-LDH/MoS_2_ hybrid material were investigated in detail. Then MoS_2_, NiCo-LDH and NiCo-LDH/MoS_2_ filled TPU composites were prepared by melt blending, and their effects on the flame retardant, smoke suppression properties, thermal stability and toxic gas emission of TPU were further studied.

## 2. Experimental

### 2.1. Materials

Nickel (II) nitrate hexahydrate (Ni(NO_3_)_2_·6H_2_O, AR) was supplied by Tianjin Dingshengxin Chemical Co. Ltd., Tianjin, China. Molybdenum disulfide (MoS_2_) and n-butyllithium (C_4_H_9_Li, AR) were provided by Sinopharm Group Chemical Reagent Co. Ltd., Shanghai, China. Cobalt (II) nitrate hexahydrate (Co(NO_3_)_2_∙6H_2_O, AR), 2-Methylimidazole (2-MIM, AR), ethanol absolute, methanol were purchased from Aladdin Chemical Reagent Co., Ltd., Shanghai, China. Thermoplastic polyurethane (TPU, 9380A) was bought from Germany’s bayer, Shanghai, China.

### 2.2. Synthesis of 3D Hollow NiCo-LDH

ZIF-67 was synthesized according to previous reports in the literature [21,22]. 3D hollow NiCo-LDH was fabricated using the hydrothermal method. Typically, 200 mg of ZIF-67 was dissolved in 50 mL of anhydrous ethanol and ultrasonically dispersed for 30 min, which was recorded as liquid A. Then 600 mg of nickel nitrate hexahydrate was ultrasonically dispersed in 30 mL of anhydrous ethanol, which was recorded as liquid B. Finally, mix liquid A and liquid B uniformly and then transfer them into a 100 mL Teflon-lined autoclave, and reacted at 90 °C for 2 h. After cooling to room temperature, the obtained product was washed three times with ethanol in a centrifuge, and dried in an oven at 60 °C for 10 h to obtain hollow NiCo-LDH.

### 2.3. Synthesis of 3D Hollow NiCo-LDH/MoS_2_ hybrid material

**Figure 1:**
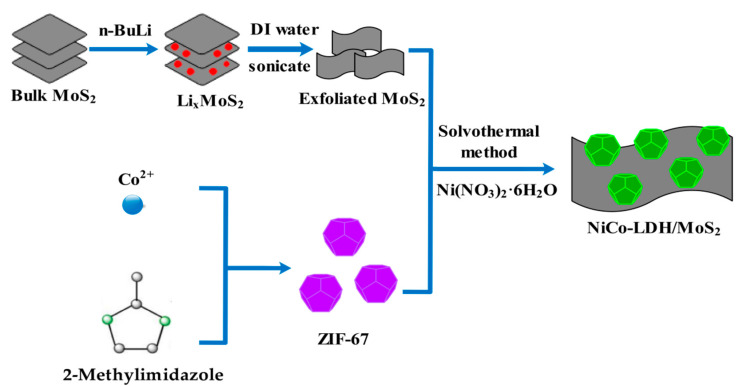
The preparation process diagram of 3D hollow NiCo-LDH/MoS_2_.

Preparation of exfoliated MoS_2_ nanosheets by Li-ion intercalation [23]. The 3D hollow NiCo-LDH/MoS_2_ hybrid material was also synthesized by the hydrothermal method. Figure 1 is the preparation process diagram of the 3D hollow NiCo-LDH/MoS_2_ hybrid material.

### 2.4. Synthesis of TPU Composites

Under the mixing conditions of 180 °C and 30 rpm, 58 g of TPU was added to the mixer, and then the flame retardants with a mass fraction of 2 wt% were blended into the TPU matrix and stirred for 10 min. The specific formulations of TPU composites are shown in Table 1. Afterwards, the prepared TPU composites were put into a tablet press (180 °C, 10 MPa), and the TPU composites were hot-pressed for 10 min and cold-pressed for 3 min to obtain a size of 100 mm × 100 mm × 3 mm of TPU composites.

### 2.5. Characterization

X-ray diffraction (XRD) was recorded on an X-ray diffractometer equipped with Cu-Kα tube and Ni filter (λ = 0.1542 nm), and the diffraction angle (2θ) ranged from 5° to 80°. Fourier transform infrared (FTIR) spectra of the prepared samples were performed by a Nicolet 6700 FTIR spectrophotometer in the range of 4000 cm^−1^ to 400 cm^−1^. The morphology of the prepared samples was observed using a scanning electron microscope (SEM), accompanied by an accelerating voltage of 9 kV. Transmission electron microscopy-energy dispersive spectrometer (TEM-EDS) images were performed with a JEM-2100Plus instrument at 200 KV. X-ray photoelectron spectroscopy (XPS) characterizes the elemental chemical states of the prepared samples. The thermal stability of the samples was tested using a thermogravimetric analyzer (TGA). About 10.0 mg of the sample was placed in an alumina crucible and heated from 40 °C to 800 °C at a linear heating rate of 20 °C/min, setting the N_2_ flow rate to 20 mL/min. The combustion tests were carried out on a cone calorimeter according to the ISO 5660 test standard. All TPU composites (100 mm × 100 mm × 3 mm) were wrapped with aluminum foil and measured under an external heat flux of 50 kW/m^2^. Analysis of pyrolysis products by Thermogravimetric-Fourier transform infrared spectrometer (TG-FTIR).

## 3. Results and Discussions

### 3.1. Characterization of Hollow NiCo-LDH and Its hybrid material

**Figure 2:**
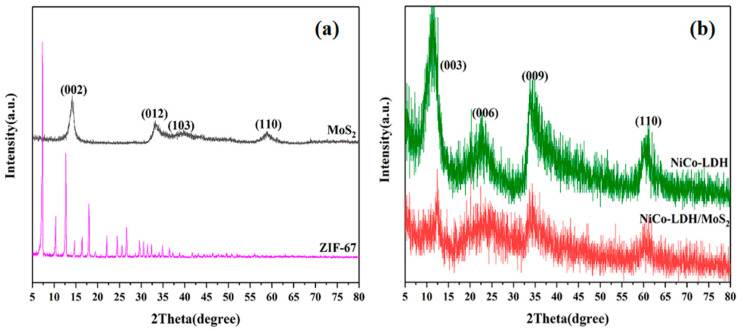
XRD patterns of MoS_2_, ZIF-67 (**a**) and NiCo-LDH, NiCo-LDH/MoS_2_ (**b**).

The structural information of the prepared samples was analyzed by XRD, as shown in Figure 2. As demonstrated in Figure 2a, the XRD pattern of MoS_2_ exhibits an obvious peak at 2θ = 14.2°, corresponding to the (002) plane. In addition, the characteristic peaks of ZIF-67 are in agreement with the previously reported work [21]. For NiCo-LDH, reflections at 2θ = 11.3°, 22.9°, 34.1° and 60.7° can be indexed as (003), (006), (009), and (110) diffraction peaks of NiCo-LDH, respectively, while the diffraction peaks are sharper, indicating that NiCo-LDH has a good crystalline form [24]. It can be seen from the figure that the NiCo-LDH/MoS_2_ hybrid material has similar characteristic peaks to NiCo-LDH, and there is no diffraction peak corresponding to MoS_2_, indicating a high dispersion state of MoS_2_ in the hybrid material. This is mainly attributed to the loss of face-to-face stacking structure of MoS_2_ nanosheets for the growth of NiCo-LDH on MoS_2_ surfaces [25]. Compared with NiCo-LDH, the diffraction peaks of NiCo-LDH/MoS_2_ are blunt, which is caused by the disorder of the stacked structure between NiCo-LDH and MoS_2_ nanosheets.

**Figure 3:**
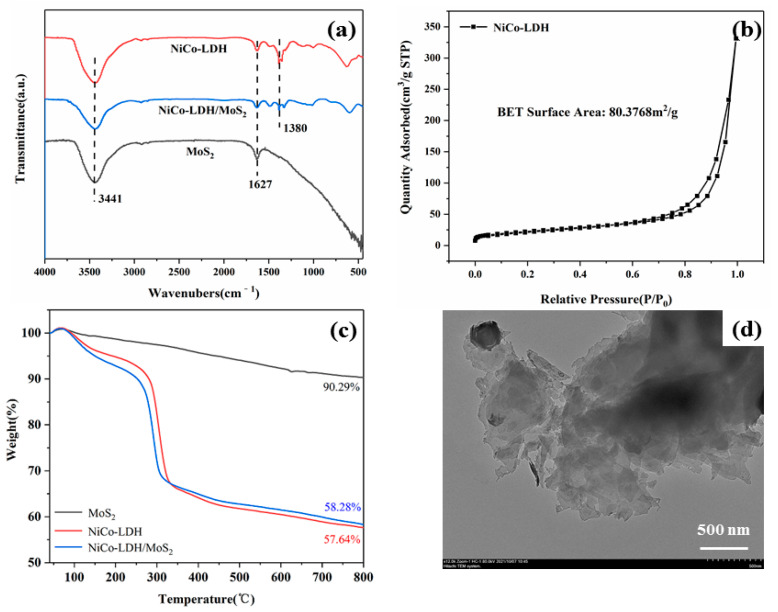
FTIR spectra of MoS_2_, NiCo-LDH and NiCo-LDH/MoS_2_ (**a**); N_2_ adsorption−desorption isotherms of NiCo-LDH (**b**); TG curves of MoS_2_, NiCo-LDH and NiCo-LDH/MoS_2_ (**c**); TEM image of MoS_2_ (**d**).

Figure 3a shows the FTIR spectra of MoS_2_, NiCo-LDH and NiCo-LDH/MoS_2_. It can be seen from Figure 3a that the broad absorption peak at 3443 cm^−1^ of the three materials originates from the stretching vibration of the -OH group. The characteristic peak at 1627 cm^−1^ is ascribed to the bending vibration of water molecules. For NiCo-LDH, the stretching vibration of NO_3_^−^ is also detected at around 1380 cm^−1^. Due to the lattice vibration of metal-O, NiCo-LDH exhibits characteristic absorption peaks at 500–800 cm^−1^ [26]. The BET surface area of hollow NiCo-LDH was detected by N_2_ adsorption/desorption isotherm, as shown in Figure 3b. Qin et al. reported that the BET surface area of solid NiCo-LDH is about 34 m^2^/g [27]. Interconnected NiCo-LDH nanosheets are loosely stacked on the precursor surface to form a highly porous structure, therefore NiCo-LDH has a high BET surface area of 80.3768 m^2^/g, which helps the NiCo-LDH to form more interfaces with the polymer matrix, thus improving the interaction between the two. Figure 3c shows the TG curves of MoS_2_, NiCo-LDH and NiCo-LDH/MoS_2_. It is noted that MoS_2_ nanosheets have high thermal stability, and the char yield of MoS_2_ is 90.29%. NiCo-LDH undergoes three thermal degradation processes by which the loss of interlayer H_2_O, decomposition of metal hydroxide, and collapse of metal organic framework. NiCo-LDH/MoS_2_ and NiCo-LDH have similar thermal decomposition trends, which might be attributed to the lower content of MoS_2_ in the hybrid material. In addition, the char yield of NiCo-LDH/MoS_2_ (58.28%) is higher than that of NiCo-LDH (57.64%), indicating that NiCo-LDH/MoS_2_ has better high temperature thermal stability. In Figure 3d, MoS_2_ nanosheets exhibit a sheet-like structure. The dark parts in the sample can be attributed to the partial aggregation of MoS_2_ nanosheets.

**Figure 4:**
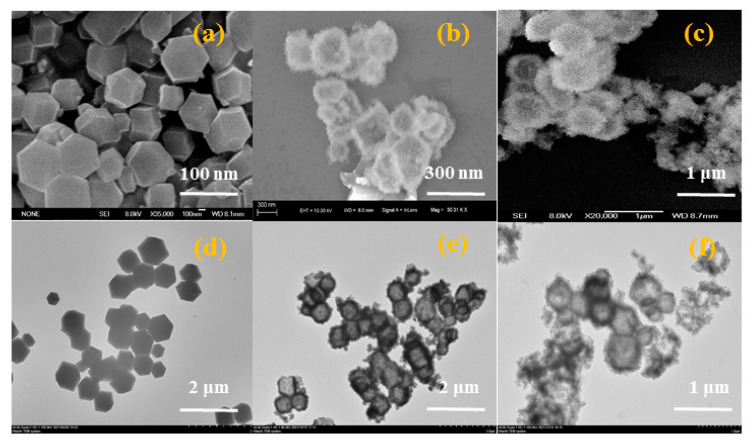
SEM and TEM images of ZIF-67 (**a**,**d**),NiCo-LDH (**b**,**e**) and NiCo-LDH/MoS_2_ (**c**,**f**).

Morphologies of ZIF-67, NiCo-LDH and NiCo-LDH/MoS_2_ were observed by TEM and SEM, as shown in Figure 4. It can be seen from Figure 4a,d that the precursor ZIF-67 exhibits a solid regular dodecahedron morphology with uniform size (about 100 nm), and its surface is considerably smooth. As shown in Figure 4b,e, NiCo-LDH still maintains the morphology of the precursor and has a hollow interior, and NiCo-LDH nanosheets grown on the surface of the ZIF-67 precursor. From Figure 4c, it can be seen that the NiCo-LDH/MoS_2_ hybrid material fails to detect MoS_2_, which may be caused by the complete coverage of MoS_2_ nanosheets by NiCo-LDH.

Figure S1 gives the EDS spectrum and plan scan image of NiCo-LDH/MoS_2_. It can be seen from Figure S1 that Co, Ni and O elements are uniformly distributed on the ZIF-67-derived hollow dodecahedral framework. At the same time, the detected Mo element also confirms the existence of MoS_2_, indicating the successful preparation of NiCo-LDH/MoS_2_ hybrid material.

**Figure 5:**
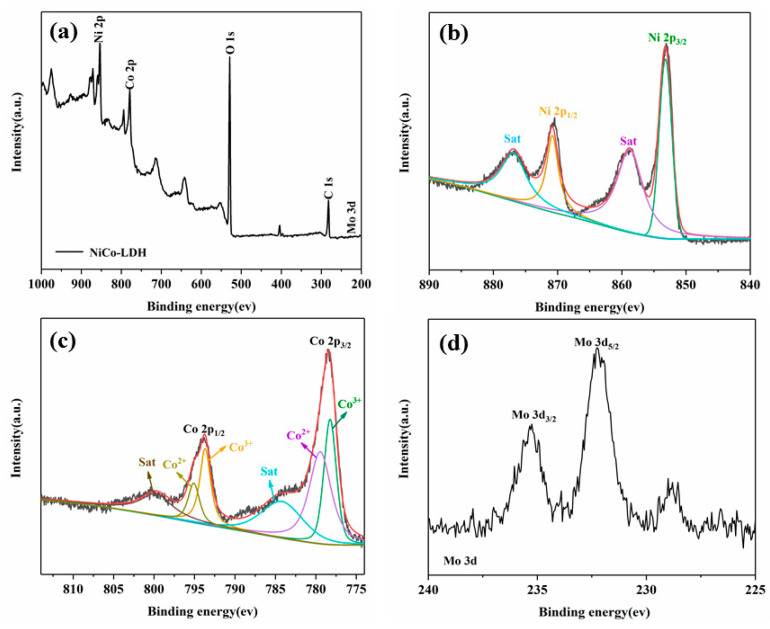
XPS survey spectrum of NiCo-LDH/MoS_2_ (**a**), high-resolution XPS spectra of Ni 2p, Co 2p and Mo 3d for NiCo-LDH/MoS_2_ (**b**–**d**).

XPS was used to test the chemical composition of NiCo-LDH/MoS_2_ and the valence states of the elements, and the results are presented in Figure 5. Figure 5a is the XPS survey spectrum of NiCo-LDH/MoS_2_, indicating the presence of C, O, Ni, Co and Mo elements of hybrid material. This further proves the successful hybridization of NiCo-LDH and MoS_2_. In the high-resolution Ni 2p spectrum, the peaks at 853.2 ev and 870.8 ev can be attributed to Ni 2p_3/2_ and Ni 2p_1/2_. Another two peaks of 858.8 ev and 877.2 ev correspond to the satellite shake-up peaks of Ni 2p_3/2_ and Ni 2p_1/2_, respectively [27]. These peaks prove that the valence state of the Ni element is divalent. As shown in Figure 4c, the binding energies of Co^2+^ 2p_3/2_ and Co^2+^ 2p_1/2_ at fitting peaks 779.3 ev and 795.2 ev, while the binding energy peaks at 778.2 ev and 793.7 ev refer to Co^3+^ 2p_3/2_ and Co^3+^ 2p_1/2_ [28].

### 3.2. CCT Analysis

It has been demonstrated that the results of the CCT correlate well with the results obtained in the large-scale fire tests and can be used to predict the burning behavior of polymers in real fires [29]. Therefore, the effects of MoS_2_, NiCo-LDH and NiCo-LDH/MoS_2_ on the flame retardant and smoke suppression properties of TPU composites were further investigated by the cone calorimeter.

**Figure 6:**
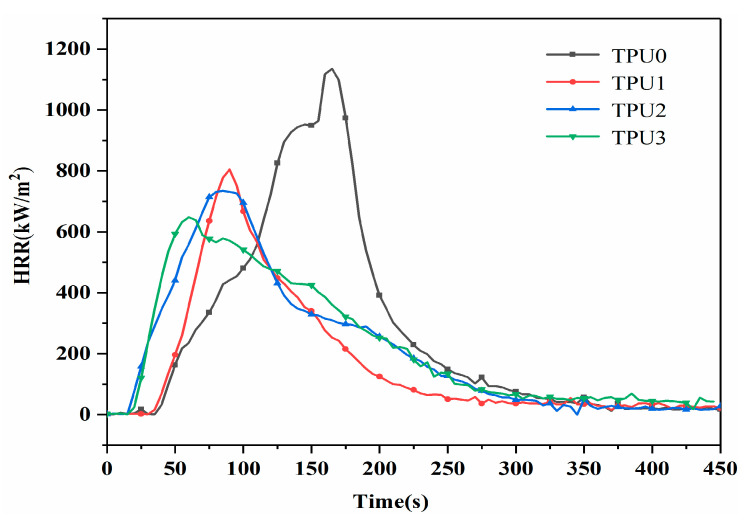
Heat release rate curves of TPU composites.

Heat release rate (HRR) is an important indicator to describe the fire hazard of polymers and can predict the behavior of polymers under real combustion conditions [30]. The HRR results for pure TPU and TPU composites are given in Figure 6 and Table 2. In Figure 6, pure TPU burns fiercely after being ignited and has the highest peak heat release rate (PHRR) of 1135 kW/m^2^. The addition of 2 wt% NiCo-LDH and MoS_2_ nanosheets slightly decreases the PHRR of TPU composites to 804 kW/m^2^ and 734 kW/m^2^, respectively, indicating that MoS_2_ and NiCo-LDH nanosheets can inhibit the heat release of TPU composites and improve the flame retardancy of TPU composites to a certain extent. It is worth noting that the PHRR value of NiCo-LDH/MoS_2_ filled TPU composite is lower than that of single MoS_2_ or NiCo-LDH filled TPU composites, indicating that NiCo-LDH and MoS_2_ have synergistic flame retardant effect. On the one hand, the transition metals nickel and cobalt in NiCo-LDH have catalytic carbonization effects. Coke can form a barrier effect on the polymer surface, slow down heat and mass transfer between the gas phase and the condensed phase, and protect the underlying material from further combustion [31]. On the other hand, two-dimensional MoS_2_ nanosheets have nano-barrier effect, which can hinder the release of volatile products including hydrocarbons, so that less volatile products form fuel into the combustion zone, thereby reducing the heat release rate [32]. It is not difficult to see from Figure 6 that the ignition time of TPU composites is longer than that of pure TPU, which is related to the decomposition of MoS_2_ and NiCo-LDH at low temperatures. Of note, The addition of MoS_2_ nanosheets or NiCo-LDH/MoS_2_ shortens the ignition time of TPU composites, which is attributed to the early decomposition of MoS_2_ nanosheets or NiCo-LDH/MoS_2_.

**Figure 7:**
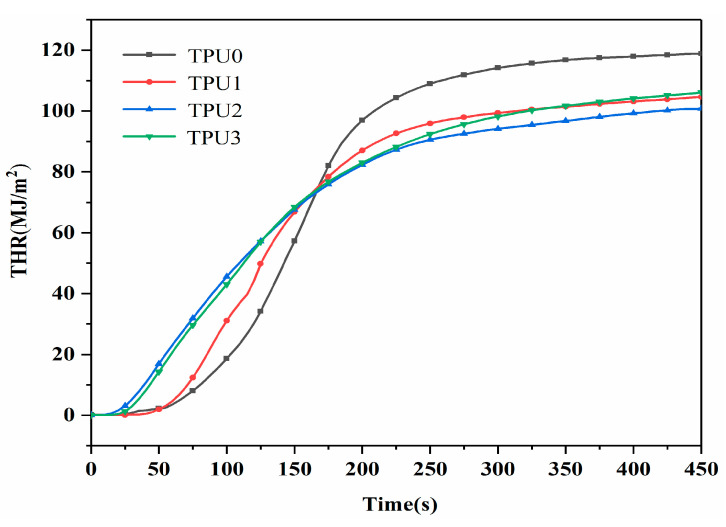
Total heat release curves of TPU composites.

Figure 7 exhibits the total heat release (THR) curves of pure TPU and TPU composites. Pure TPU has the highest THR value of 118.8 MJ/m^2^. After the incorporation of 2 wt% NiCo-LDH and MoS_2_ separately, the THR values of TPU composites are decreased to 104.6 MJ/m^2^ and 100.7 MJ/m^2^, respectively, this is mainly because the uniformly dispersed NiCo-LDH and MoS_2_ nanosheets in the TPU matrix can inhibit the release of combustible gases during the combustion process, thereby promoting carbonization. Meanwhile, NiCo-LDH will release water vapor and reduce the surface temperature of TPU substrate during the combustion process, so as to achieve better flame retardant effect [33]. However, when incorporating 2 wt% NiCo-LDH/MoS_2_ hybrid material into TPU, the THR value of TPU3 increased to 106.1 MJ/m^2^, which may be due to the combination of MoS_2_ and NiCo-LDH further reducing the exfiltration rate of combustible gas, leading to more complete oxidative combustion of combustible volatiles such as hydrocarbons and thus generating more heat.

**Figure 8:**
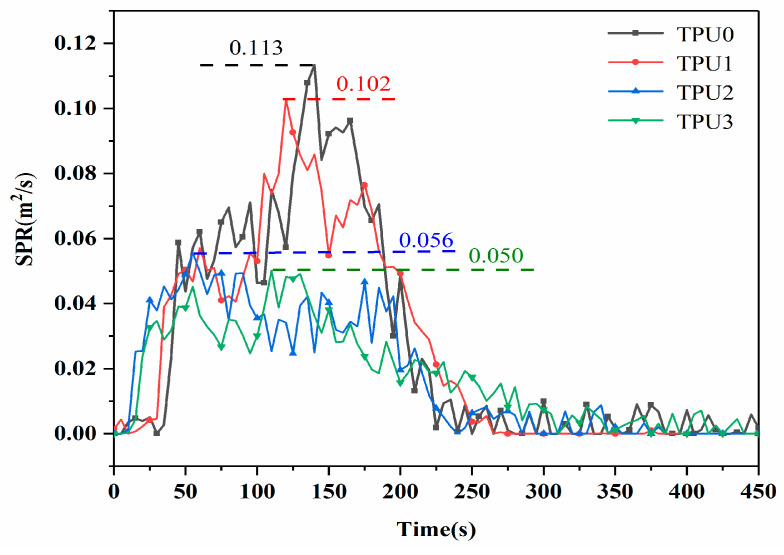
Smoke production rate curves of TPU composites.

The amount of heavy smoke released during the combustion process is an important parameter to evaluate the fire hazard of TPU. The SPR (smoke production rate) curves of pure TPU and its composites are shown in Figure 8. The PSPR (peak smoke production rate) value of pure TPU reaches 0.113 m^2^/s, indicating the highest smoke emission. Nevertheless, the addition of MoS_2_ has little effect on the PSPR value of the TPU composite, indicating that MoS_2_ alone could not achieve satisfactory smoke suppression effect. In contrast, the PSPR value of NiCo-LDH filled TPU2 is further reduced to 0.056 m^2^/s, which is reduced by 50.4% as compared to that of pure TPU. It is ascribed that the transition metals Ni and Co have the effect of catalytic carbonization, and the formed carbon layer can reduce the combustible gas and smoke-forming materials in the gas phase. In addition, the porous structure of 3D hollow NiCo-LDH can absorb organic volatiles generated by the thermal decomposition of TPU, which are the main source of smoke particles [34]. When TPU composite is reinforced with NiCo-LDH/MoS_2_ hybrid material, its PSPR value is further reduced to 0.05 m^2^/s, clearly revealing the significant enhancement of smoke suppression performance of TPU composites. This is mainly attributed to NiCo-LDH and MoS_2_ decomposed nickel, cobalt and molybdenum compounds can catalyze the formation of carbon, which can suppress the smoke production rate of TPU composites [32].

**Figure 9:**
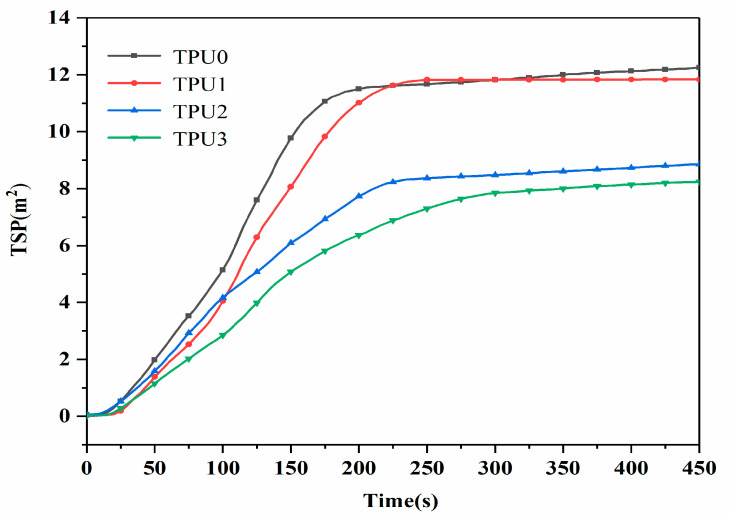
Total smoke production curves of TPU composites.

The TSP (total smoke production) curves for pure TPU as well as TPU composites are given in Figure 9. Obviously, pure TPU releases the most smoke during combustion, with a TSP value as high as 12.3 m^2^. It is worth noting that the TSP value of TPU1 is 11.9 m^2^, which is basically the same as that of pure TPU. Compared with pure TPU, the TSP value of TPU2 is 8.8 m^2^, corresponding to a decrease of 28.5%. This can be explained that organic volatile is the main source of smoke particles, and the presence of NiCo-LDH makes TPU molecules more retained in the condensed phase without being converted into an organic volatile [35]. The TSP value of the TPU composite with NiCo-LDH/MoS_2_ hybrid material is further reduced to 8.2 m^2^, corresponding to a 33.3% reduction compared to pure TPU. The above results indicate that the combination of two additives imparts better smoke suppression to TPU.

### 3.3. Thermal Stability Analysis

TGA is a widely used technique to rapidly evaluate the thermal stability of materials, and can also reveal the thermal degradation behavior of polymers at different temperatures [36]. To profoundly understand the influence of MoS_2_, NiCo-LDH and NiCo-LDH/MoS_2_ hybrid material on the thermal stability of TPU composites, the thermal oxidative degradation behavior and carbon residues of different TPU composites were compared using TGA.

**Figure 10:**
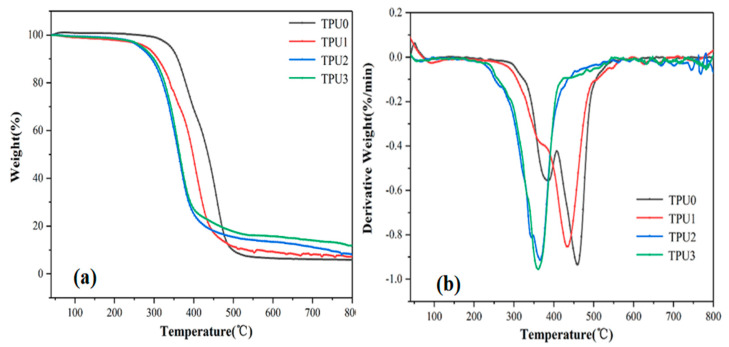
TG (**a**) and DTG (**b**) curves of pure TPU and TPU composites.

The TG and DTG profiles for pure TPU and TPU composites under N_2_ atmosphere are displayed in Figure 10, and the related data are summarized in Table 3. From Figure 10b, it can be seen that pure TPU mainly presents two decomposition stages. More precisely, the first decomposition stage corresponds to the removal of CO_2_, and the second decomposition stage is mainly attributed to the dehydration carbonization reaction [37]. In contrast, TPU composites exhibit only one thermal decomposition stage, the rapid decomposition stage of TPU composites occurs between 250 °C and 450 °C. From Figure 10a, it can be easily observed that the initial decomposition temperature (T_−5%_, temperatures at 5% weight loss) of the TPU composites is lower than that of pure TPU, which is mainly attributed to the early degradation of MoS_2_ and NiCo-LDH. In addition, the T_−5%_ of TPU3 is 6 °C higher than that of TPU2, demonstrating that the NiCo-LDH/MoS_2_ hybrid material improved the thermal stability of TPU composites. As can be seen, the char yields of TPU1 and TPU2 at 800 °C are 7.93% and 8.02%, which are 1.36 and 1.37 times than that of pure TPU, respectively. The enhanced char yields may be due to the addition of MoS_2_ or NiCo-LDH, implying the formation of effective barrier layers in the TPU composites. Furthermore, the char yield of TPU3 reaches 11.87% at 800 °C. demonstrating that NiCo-LDH and MoS_2_ nanosheets jointly promote the improvement of char yield of TPU composite from the above analysis, the catalytic carbonization effect of NiCo-LDH/MoS_2_ hybrid material can enhance the thermal stability of TPU composites, which is beneficial to improve the fire safety of TPU composites [38].

### 3.4. Char Residue Analysis

In order to study the flame retardant mechanism of NiCo-LDH/MoS_2_ in the condensed phase, the digital photos of carbon residues of TPU composites were firstly investigated. Figure S2 gives digital photos of carbon residues of pure TPU of TPU composites after the cone calorimeter test. It can be clearly seen from Figure S2 that the pure TPU burns very completely, and there is no remaining carbon residue. For TPU1 added with MoS_2_, the carbon layer is not complete, which also leads to the inability of TPU1 to effectively suppress mass and heat transfer during the combustion process. Although the carbon residue of TPU2 covers the entire aluminum foil, the carbon residue is loose and fragile. On the contrary, by incorporating NiCo-LDH/MoS_2_ hybrid material into TPU, the amount of carbon residual increases significantly, and the carbon layer is thicker and harder, indicating that NiCo-LDH/MoS_2_ hybrid material has excellent catalytic carbonization effect.

**Figure 11:**
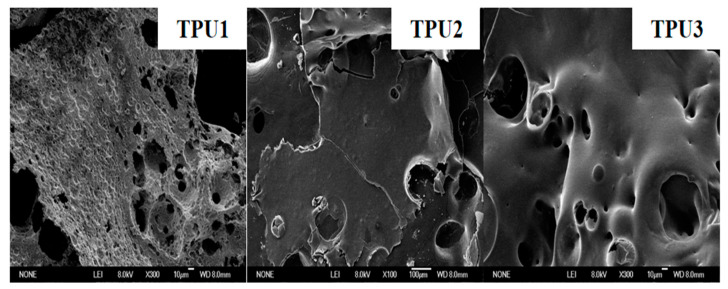
SEM images of the carbon residues of TPU composites.

The SEM images of the carbon residues of the TPU composite are shown in Figure 11. It is clear that there are obvious cracks and a large number of holes in the carbon residual of TPU1. When NiCo-LDH is added to TPU, the carbon residue of TPU2 has a more continuous structure, but there are still some cracks and holes. In comparison, the application of NiCo-LDH/MoS_2_ hybrid material in TPU gives a denser and less porous carbon residue. The dense carbon layer structure can not only effectively inhibit the transfer of heat and volatiles, but also protect the underlying polymer, thereby significantly improving the thermal stability and flame retardant properties of TPU composites [39].

**Figure 12:**
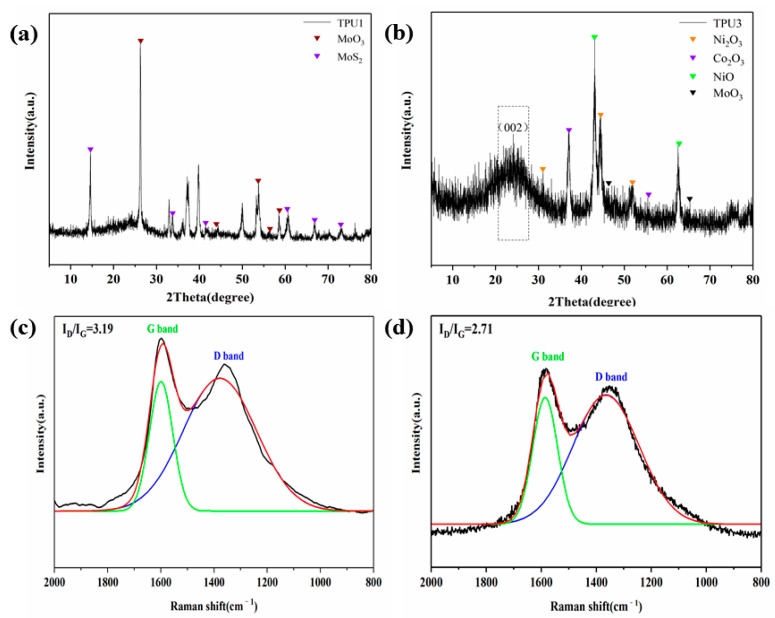
XRD patterns of carbon residues of TPU1 (**a**) and TPU3 (**b**); Raman spectra of TPU1 (**c**) and TPU3 (**d**).

The carbon residues of TPU1 and TPU3 were analyzed by XRD and Raman tests, and the results are shown in Figure 12. It can be found in Figure 12a that diffraction peaks of MoO_3_ and MoS_2_ appeared in the XRD pattern of the carbon residue of TPU1. As shown in Figure 12b, the XRD pattern of the carbon residue of TPU3 not only has characteristic peaks of metal oxides such as MoO_3_, NiO, Ni_2_O_3_ and Co_2_O_3_, but also has a diffraction peak of graphite crystallite around 2θ = 25°. The degree of graphitization of carbon residues was determined by Raman spectra, as shown in Figure 12c,d. It is clear from both Raman spectra that obvious peaks are observed at 1360 cm^−1^ and 1598 cm^−1^, representing the D and G bands, respectively. The area ratio of the D band and G band (I_D_/I_G_) is widely used to determine the degree of graphitization of carbon residues. The lower the value of I_D_/I_G_, the higher the degree of graphitization of the carbon residues [40]. The I_D_/I_G_ value of TPU3 is 2.71, lower than that of TPU1 (3.19), which indicates that the addition of NiCo-LDH/MoS_2_ is beneficial to improving the degree of graphitization of carbon residues.

### 3.5. Thermal Decomposition Products Analysis

In order to obtain information about the variation of gaseous products with temperature during the thermal decomposition of TPU composites, 3D TG-FTIR spectra of TPU0 and TPU3 are given in Figure S3. As shown in Figure S3, the thermal decomposition process of TPU0 and TPU3 is similar, which indicates that the addition of NiCo-LDH/MoS_2_ hybrid material has little effect on the inherent properties of TPU. In addition, it can be obviously seen that the temperature at which TPU3 releases thermal decomposition products is lower than that of TPU0, which is primarily because the addition of NiCo-LDH/MoS_2_ hybrid material makes the initial decomposition temperature of TPU composite earlier.

**Figure 13:**
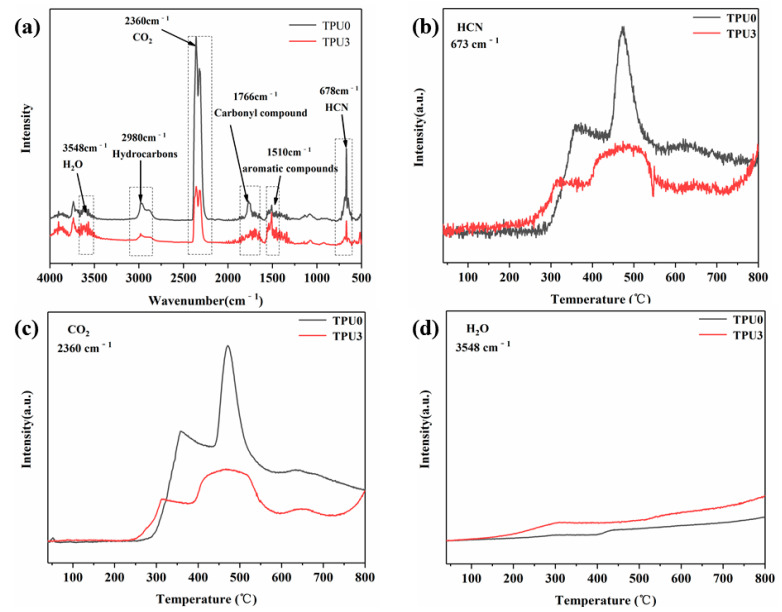
FTIR spectra of TPU0 and TPU3 pyrolysis products at maximum decomposition rate (**a**), the absorbance intensities of HCN (**b**), CO_2_ (**c**) and H_2_O (**d**) of TPU0 and TPU3.

Figure 13a presents the FTIR spectrum of the pyrolysis products of TPU0 and TPU3 at the maximum decomposition rate. The characteristic peak at 3548 cm^−1^ is ascribed to the vibration of the O-H bond in H_2_O. The absorption peak appearing at 2980 cm^−1^ is assigned to the symmetrical stretching vibration of the C-H bond in hydrocarbons. The peaks at 2360 cm^−1^ and 1766 cm^−1^ are typical absorption peaks of CO_2_ and carbonyl compounds, respectively. The absorption peaks of aromatic hydrocarbons and HCN are located at 1510 cm^−1^ and 678 cm^−1^, respectively. As shown in Figure 13b,c, the presence of NiCo-LDH/MoS_2_ hinders the release of HCN and CO_2_, which is mainly attributed to the formation of high-quality carbon residues and the barrier effect of MoS_2_ nanosheets [41]. From Figure 13d, it is observed that the amount of H_2_O released in the pyrolysis product of TPU3 is significantly higher than that of pure TPU, which is beneficial to diluting the combustible gas.

### 3.6. Flame Retardant Mechanism

Based on the flame retardant properties and condensed phase-gas phase analysis of TPU/NiCo-LDH/MoS_2_ composite above, a possible flame retardant mechanism was proposed. In the condensed phase: (1) The transition metals Ni, Co and Mo have the effect of catalytic carbonization. The formed carbon layer can block the transfer of heat and combustible gas between the combustion zone and the TPU matrix, and protect the unburned TPU matrix. (2) During combustion, MoS_2_ and NiCo-LDH act as physical barriers, slowing down the escape of combustible, hindering the permeation of oxygen and inhibiting the exudation of toxic substances. (3) The metal oxides generated by the decomposition of NiCo-LDH/MoS_2_ not only improve the degree of graphitization of the carbon layer, but also can absorb the flue gas generated during the combustion process [34]. In the gas phase, NiCo-LDH releases non-combustible gases (H_2_O, CO_2_) that can dilute the concentration of combustible gases to some extent.

## 4. Conclusions

In conclusion, 3D hollow NiCo-LDH was assembled on MoS_2_ nanosheets by the principle of electrostatic self-assembly to form 3D hollow NiCo-LDH/MoS_2_ hybrid material. Characterization of the structure and morphology of the NiCo-LDH/MoS_2_ by XRD, FTIR, SEM, TEM, BET and XPS. Then the NiCo-LDH/MoS_2_ was mixed with TPU by melt blending. With the addition of NiCo-LDH/MoS_2_, PHRR, PSPR and TSP values of the obtained TPU composite were remarkably decreased by 42.9%, 55.7% and 33.3%. Meanwhile, the TPU composite filled with NiCo-LDH/MoS_2_ hybrid material had higher char yield and thermal stability. In addition, SEM, XRD and Raman spectroscopy revealed that the NiCo-LDH/MoS_2_ filled TPU composite has dense carbon residue with enhanced graphitization degree, which is able to protect the underlying TPU matrix. TG-FTIR results showed NiCo-LDH/MoS_2_ hybrid material also exhibits excellent toxic gas (HCN) suppression performance. In summary, the catalytic carbonization effect and dilution effect of NiCo-LDH, and the barrier effect of MoS_2_ nanosheets enable TPU composites with excellent flame retardancy, thermal stability and toxic gas suppression ability.

## Figures and Tables

**Table 1:** TPU composites formula table.

Sample Code	TPU (wt%)	MoS_2_ (wt%)	NiCo-LDH (wt%)	NiCo-LDH/MoS_2_ (wt%)
TPU0	100	0	0	0
TPU1	98	2	0	0
TPU2	98	0	2	0
TPU3	98	0	0	2

**Table 2:** Cone calorimeter data of TPU composites.

Sample Code	PHRR kW/m^2^	THR MJ/m^2^	PSPR m^2^/s	TSP m^2^
TPU0	1135	118.8	0.113	12.3
TPU1	804	104.6	0.102	11.9
TPU2	734	100.7	0.056	8.8
TPU3	648	106.1	0.050	8.2

**Table 3:** Thermogravimetry data of pure TPU and TPU composites.

Sample Code	T_−5%_ (°C)	T_max_ (°C)	Char Yield (%)
TPU0	333	454	5.85
TPU1	317	432	7.93
TPU2	264	366	8.02
TPU3	270	360	11.87

## Data Availability

The data presented in this study are available on request from the corresponding author.

## Supplementary Materials

The following are available online at https://www.mdpi.com/article/10.3390/polym14112204/s1, Figure S1: EDS spectrum (a) and plan scan image (b) of NiCo-LDH/MoS_2_; Figure S2: Digital photographs of the char residues of TPU composites; Figure S3: TG-FTIR spectra of thermal decomposition products of TPU0(a) and TPU3(b).
