# Supplementary material for: Cooperative Effect of ZIF-67-Derived Hollow NiCo-LDH and MoS2 on Enhancing the Flame Retardancy of Thermoplastic Polyurethane
Source: Polymers (Basel). 2022 May 29;14(11):2204. doi: 10.3390/polym14112204 (PMC9183196; doi:10.3390/polym14112204)
Supplement: Supplementary file 1 [file polymers-14-02204-s001.zip › polymers-1745988-supplementary.pdf]

Synergistic effect of ZIF-67-derived hollow NiCo-LDH and MoS_2 on enhancing the flame retardancy of thermoplastic polyurethane

Yi Qian[1*], Wenyuan Su[1], Long Li[2*], Haoyan Fu[1], Jiayin Li[2], Yihao Zhang[2], Rongmin Zhao[3], Qingjie Guo[4] and Jingjing Ma[4]

1 College of Chemical Engineering, Qingdao University of Science and Technology, Qingdao 266042, China

2 College of Environment and Safety Engineering, Qingdao University of Science and Technology, Qingdao 266042, China

3. Qingdao University of Science and Technology Library, Qingdao 266042, China

4. State Key Laboratory of High-Efficiency Utilization of Coal and Green Chemical Engineering(Ningxia University, Yinchuan 750021, China)

* E-mail: qianyi1962@126.com; lilongyln@yeah.net

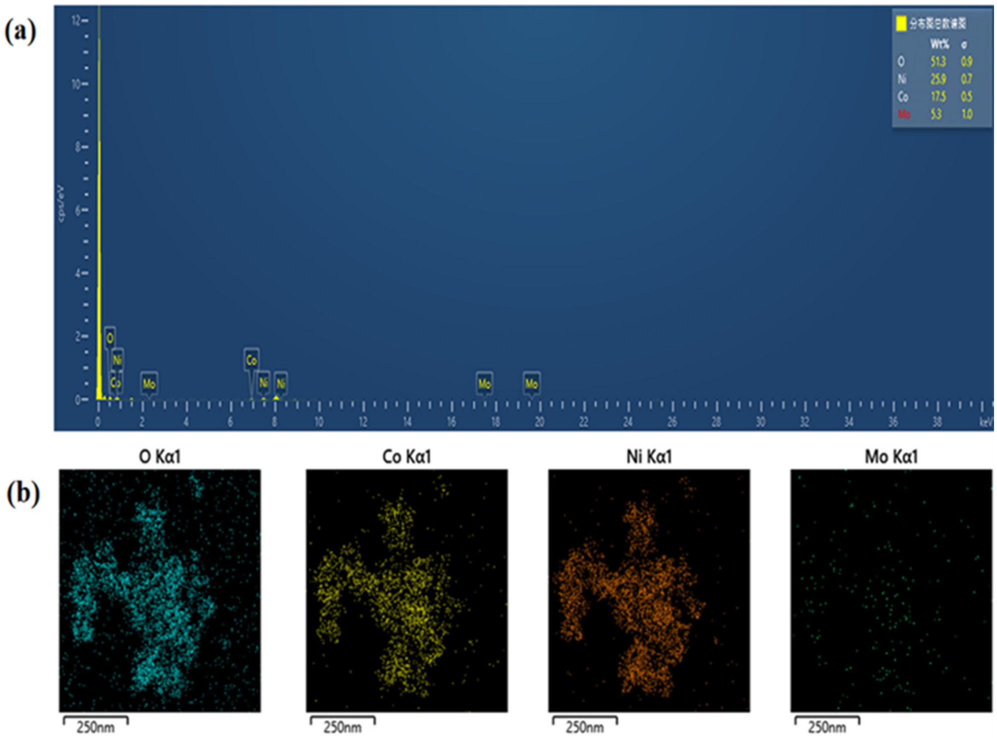

Figure S1. EDS spectrum (a) and plan scan image (b) of NiCo-LDH/MoS_2

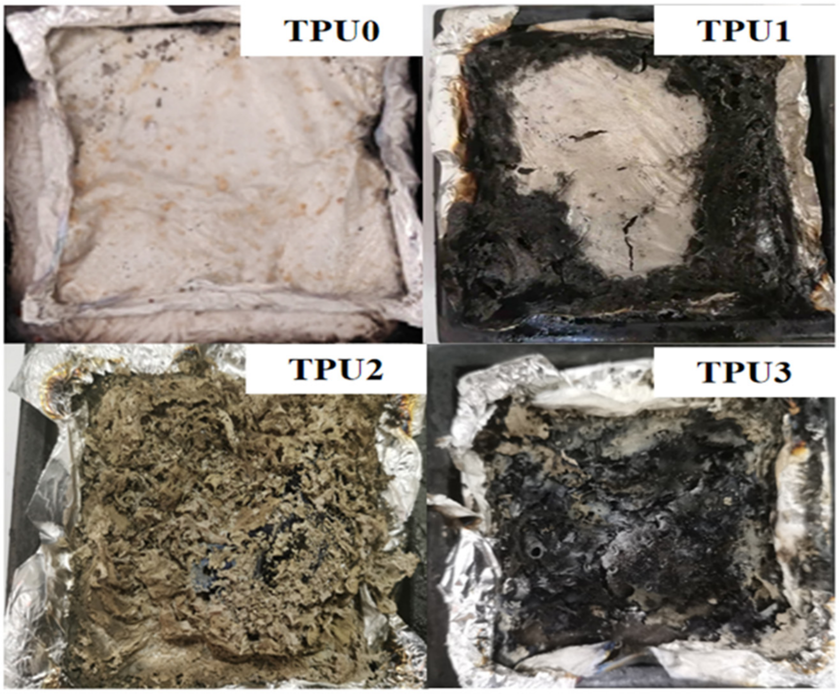

Figure S2. Digital photographs of the char residues of TPU composites

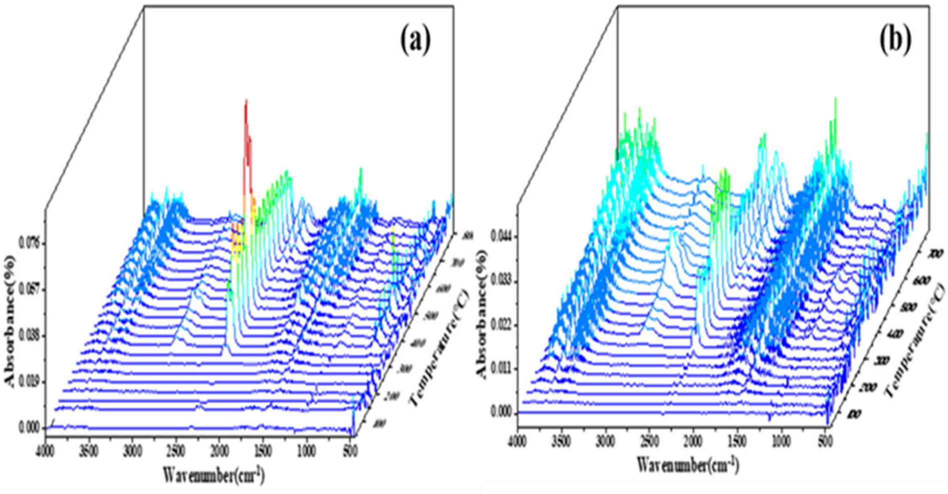

Figure S3. TG-FTIR spectra of thermal decomposition products of TPU0(a) and TPU3(b)
